# Lowering fasting blood glucose with non‐dialyzable material of cranberry extract is dependent on host genetic background, sex and diet

**DOI:** 10.1002/ame2.12291

**Published:** 2022-11-20

**Authors:** Fatima Amer‐Sarsour, Rana Tarabeih, Itzhak Ofek, Fuad A. Iraqi

**Affiliations:** ^1^ Department of Clinical Microbiology and Immunology Sackler Faculty of Medicine, Tel‐Aviv University Tel‐Aviv Israel

**Keywords:** chow diet (CHD), collaborative cross (CC) mouse model, fasting blood glucose (FBG), high‐fat diet (HFD), non‐dialyzable material (NDM) of cranberry extract, type 2 diabetes (T2D)

## Abstract

**Background:**

Type 2 diabetes (T2D) is a polygenic metabolic disease, characterized by high fasting blood glucose (FBG). The ability of cranberry (CRN) fruit to regulate glycemia in T2D patients is well known. Here, a cohort of 13 lines of the genetically diverse Collaborative Cross (CC) mouse model was assessed for the effect of non‐dialyzable material (NDM) of cranberry extract in lowering fasting blood glucose.

**Methods:**

Eight‐week‐old mice were maintained on either a standard chow diet (control group) or a high‐fat diet (HFD) for 12 weeks, followed by injections of intraperitoneal (IP) NDM (50 mg/kg) per mouse, three times a week for the next 6 weeks. Absolute FBG (mg/dl) was measured bi‐weekly and percentage changes in FBG (%FBG) between weeks 0 and 12 were calculated.

**Results:**

Statistical analysis showed a significant decrease in FBG between weeks 0 and 12 in male and female mice maintained on CHD. However, a non‐significant increase in FBG values was observed in male and female mice maintained on HFD during the same period. Following administration of NDM during the following 6 weeks, the results show a variation in significant levels of FBG lowering between lines, male and female mice and under the different diets.

**Conclusion:**

The results suggest that the efficacy of NDM treatment in lowering FGB depends on host genetic background (pharmacogenetics), sex of the mouse (pharmacosex), and diet (pharmacodiet). All these results support the need for follow‐up research to better understand and implement a personalized medicine approach/utilization of NDM for reducing FBG.

## Graphical abstract

The total experimental period of this study was 18 weeks during which mice were maintained on either HFD (42% fat) challenge or on standard CHD (18% fat). Data analysis presented based on two different periods of the study: the first period (Pre‐treatment period), followed by the second period when cranberry extract treatment starts. The first period is required for the development of diet‐induced impaired glucose tolerance and obesity in response to diet during weeks 0–12 of the experiment (8–20 weeks old). During the experiment period, measurements of body weight and glucose tolerance ability were recorded at different time points, as shown in the graphic abstract.

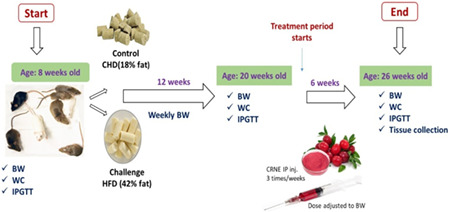

## 1 INTRODUCTION

Many studies aimed at developing glucose‐lowering drugs in patients with type 2 diabetes (T2D) have been published during the last three decades.^1^, ^2^, ^3^, ^4^ However, most pharmaceutical glucose‐lowering drugs approved for clinical use do not lead to satisfactory treatment. The safety of such drugs is a major drawback. In particular, the adverse effects of such drugs on cardiovascular complications have been of major concern.^5^ The Food and Drug Administration (FDA) has issued a request to pharma companies for more stringent measures in assessing the safety of glucose‐lowering drugs. Consequently, research has focused on alternative glucose‐lowering drugs with minimal or no side effects obtained from plant extracts. Vieira et al.^2^ have reviewed the most recent studies on novel natural sugar lowering compounds and plant extracts in the treatment of T2D. Most of these studies employed animal models (rats or mice) to evaluate the glucose‐lowering activity of the test plant extracts. Significantly, cranberry extracts were shown to reduce insulin resistance, as well as other markers of diet‐induced metabolic syndrome,^6^, ^7^ in animal models, with no apparent toxicity. Although the beneficial effect of plant extracts and in particular cranberry extracts on diet‐induced alterations of metabolic syndrome was statistically positive in these studies, in none of them was the genetic background considered. The present study was designed to determine if the effect of non‐dialyzable material (NDM) of cranberry extract on lowering the glucose levels in high fat diet (HFD)‐induced elevation of serum glucose is dependent on the genetic background. For this purpose, we employed mouse lines of defined diverse genetic backgrounds, namely Collaborative Cross (CC) mice (the next generation of mouse genetic reference populations), which provides a time and cost efficient method of dissecting the genetic variance of a specific complex trait. Full details of CC lines and their power of mapping quantitative trait loci (QTL) with host susceptibility to complex traits are presented in various publications.^8^, ^9^, ^10^, ^11^ Briefly, the CC lines were generated using a breeding scheme in which the genomes of the eight founders were combined in three out‐breeding generations, followed by inbreeding generations through sibling mating.^8^, ^9^, ^10^, ^11^, ^12^ The CC is a large panel of recombinant inbred (RI) strains derived from a genetically diverse set of eight founder strains and designed specifically for complex trait analysis and has proved a most powerful tool compared to other reported approaches. The key features of the CC in relation to gene mapping are a very large number of variants segregated in the population (there are over 36 million SNPs) and the relatively high level of recombination present compared to other mouse RI sets. Three founders of the CC (CAST/EiJ, PWK/PhJ, and WSB/EiJ) are wild derived, representing the three different subspecies *M.m castaneus*, *M.m musculus* and *M.m. domesticus*, respectively; they contribute many sequence variants not segregated among classical strains descended from *M.m. domesticus*.^8^, ^9^, ^10^, ^11^, ^12^ They allow a mapping resolution down to less than one mega base pair (Mb) by phenotyping only a relatively modest number – <100 – of CC lines.^8^, ^9^, ^12^ The choice of CC mouse lines also relies on many studies showing that this genotyped mouse population allows determination of the heritability of a test trait employing a relatively low number of mice lines.^11^ Based on these findings, in the current study, CC mice lines on the HFD were treated with cranberry extract termed NDM.^13^, ^14^, ^15^, ^16^

This unique model is a platform for research of complex traits that overcomes the limitation of existing animal models.^9^ The CC population is a powerful tool for genetic dissection of the different phenotypes. This large, multi‐parental, recombinant inbred strains panel exhibits recombinant chromosomal segments of approximately 2–5 Mb in size. The eight founder strains capture a much greater level of genetic diversity than existing RIL panels or other extant mouse genetic resource populations.^17^, ^18^, ^19^

The high molecular genomic DNA of the CC lines was genotyped in three ways: once using an array of 620 000 single nucleotide polymorphism (SNP) markers of mouse diversity,^8^ once with a mouse universal genotyping array (MUGA) using 7500 markers, and finally with a mega mouse universal genotyping array (MegaMUGA) of 77 800 markers. The updated genotype status of the population has been presented,^17^ showing the diversity of the phenotypic response of the CC lines to complex disease and their power to map quantitative trait loci (QTL) associated with a particular trait to a small genomic interval less than 0.5 cM, which consists of few genes.^20^, ^21^, ^22^, ^23^, ^24^, ^25^, ^26^, ^27^, ^28^

Recently, we have shown that the host genetics background shapes the pharmacogenetics effect of NDM on reducing body weight using the CC mouse population.^29^ Here, we present results showing that the NDM effect in lowering fasting blood glucose (FBG) levels in CC mice is significantly dependent on the genetic background of the mice (pharmacogenetics), the sex of the mice (pharmacosex) and diet (pharmacodiet). The results presented in this study support the need for follow‐up research to better understand and implement a personalized medicine approach/utilization of NDM for reducing FBG levels.

## 2 METHODS

### 2.1 Ethical statement

All experimental mice and protocols were reviewed and approved by the Institutional Animal Care and Use Committee of Tel‐Aviv University (TAU) (IACUC Approval number‐01‐17‐056).

We employed the CC mouse model which represents a powerful tool for genetic dissection of the different phenotypes.^8^, ^9^, ^10^, ^11^, ^12^, ^21^, ^30^, ^31^ Thirteen different CC mouse lines, with their TAU and international designations, and the number of founders (out of the eight founders) contributing to each line are shown in Table 1. The mice were generated and maintained at the Small Animal Facility at the Sackler Faculty of Medicine, as described in our previous studies.^8^, ^9^, ^10^, ^11^, ^12^, ^21^, ^30^, ^31^, ^32^, ^33^ Full genotype data for the CC lines developed at TAU are available at: http://mtweb.cs.ucl.ac.uk/mus/www/preCC/MEGA_MUGA/Mar2015.MEGA+MDA+MUGA/.

**TABLE 1:** List of the 13 CC mice used in the experiments, with their Tel‐Aviv University (TAU) designation and the international designations presented by the Collaborative Cross consortium (CC lines), as well as the number of contributors of the eight parental inbred strains (founders)

TAU line designation	CC line designation	Number contributors from the eight founders
IL72	CC037	6
IL111	TAU unique line	5
IL521	CC072	6
IL1912	CC051	6
IL5000	CC010	8
IL5001	CC049	8
IL5003	CC042	7
IL5004	CC039	8
IL5008	CC018	8
IL5020	CC057	8
IL5023	CC061	8
IL6009	CC035	8
IL6020	CC040	8

*Note*: CC line IL111 is a CC line unique to TAU, and has genotypes of 5 founders only, and is not yet designated under the CC system.

### 2.2 Mouse experiments

Overall, 280 mice (137 males and 143 females) from 13 different CC lines were analyzed, as shown in Table 1. TAU and CC line designations, as well the number of contributors from the eight founders, are detailed in Table 1. We used a longitudinal experiment design, where we employed a large number of CC lines (13 lines) and, except for two female lines, at least 4 mice per sex per line, to provide sufficient statistical power for calculating the mean value of trait per sex and line. At the age of 3 weeks old, mice were weaned to separate cages based on sex and CC line, with free access to standard rodent chow diet (CHD) and water. The experiment started when the mice were 8 weeks old (week zero (w0)). Throughout the following 18 weeks, the mice were maintained either on CHD or on a high‐fat diet (HFD), considered to be a Western diet, or TD. 8137 (Teklad Global, Harlan Inc.), consisting of 42.0% (Kcal) fat, 15.3% protein, and 42.7% carbohydrates. At the end of the experiment, the mice were 26 weeks old. At week 12, cranberry extract was administered intraperitoneally (see below) and measurements of FBG were taken at weeks 0, 12, and 18.

### 2.3 Measuring fasting blood glucose levels (FBG)

The FBG test is used to detect disturbances in glucose metabolism that can be linked to diabetes or metabolic syndrome. The absolute FBG (mg/dl) was assessed at the start of the experiment (w0), 12 weeks into the experiment, and 6 weeks after NDM treatment (week 18 (w18)). Mice were fasted for 6 h (6:00 AM to 12:00 AM) with free access to water. After 6 h fasting, blood glucose levels (mg/dl) were measured using a U‐RIGHT glucometer TD‐4267 (TaiDoc Technology Corporation 3 F, 5 F) as described elsewhere.^32^ Subsequently, the percentage change in FBG (%FBG) was calculated as the difference (delta) between FBG values at w0 and w12 divided by FBG value at w0.

### 2.4 Cranberry extract (CRE)

Non‐dialyzable material (NDM) of cranberry extract was obtained from a cranberry juice concentrate (50 Brix, Ocean Spray) as described previously.^13^ Briefly, the concentrated juice was dialyzed extensively in dialysis tubes of pore size 12–14 kDa (kiloDalton) against distilled water (>10× the volume of concentrated juice) with six changes over 6 days, and the non‐dialyzable material was collected, lyophilized, and chilled until use (about 6 g NDM from one‐liter concentrated juice). NDM was found to contain A‐type proanthocyanidin oligomers (PACs) of 3–6 degrees of polymerization composed of catechin units, with some gallocatechin and anthocyanin units also present, as well as quercetin derivatives.^14^ It also contains putative xyloglucans, which together with the mixed polyphenols exhibit anti‐bacterial biofilm activity. NDM extract was dissolved in distilled water to provide a dose of 50 mg/kg mouse body weight, and 200 μl was injected intraperitoneally (IP) 3 times a week for 6 weeks (from w12 until w18).

### 2.5 Statistical analysis

Statistical analysis was performed using the software package IBM SPSS Statistics version 24. One‐way ANOVA was carried out for testing the significance level of trait variation among CC lines. A *t* test was also performed to assess the changes and significance before and after diets (between weeks 0 and 12), as well after NDM treatment between weeks 12 and 18.

### 2.6 Broad sense heritability and genetic coefficient of variation

The broad‐sense heritability and genetic coefficient of variation were estimated as described in detail elsewhere.^11^ Briefly, heritability (H2) refers to the proportion of variation between individuals in a population that can be influenced by genetic factors. Here, we used the one‐way ANOVA output of the phenotypic traits to calculate the heritability (including epistatic, but not dominance effects), using the following equation:

$$H^{2}=\left(V_{g}/V_{g}+V_{e}\right)$$

where, *H* ^2^ = heritability, *V*_g_ = genetic variance, and *V*_e_ = environment variance. To evaluate the genetic dispersion of the monitored phenotypes, the genetic coefficient of variation (CV_g_) was estimated using the equation: SD_g_/Mean where SD_g_ is the broad‐sense genetic standard deviation among CC lines = V_G_^0.5^ and Mean is the mean trait value across all CC lines.

## 3 RESULTS

### 3.1 The effect of NDM on FBG values

**FIGURE 1:**
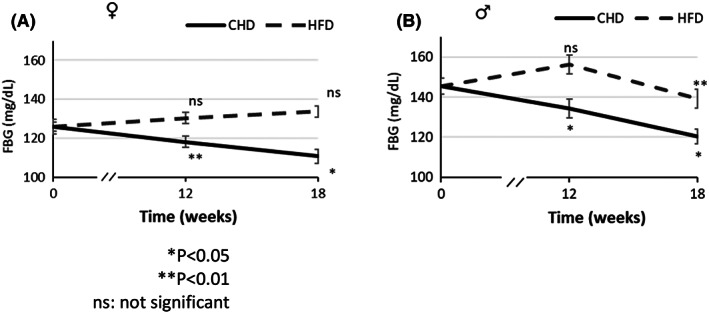
Effect of NDM on total FBG (mg/dl) in female and male mice in the different diet groups. (A) and (B) show the total FBG (mg/dl) in female and male mice respectively in HFD and CHD groups.

The absolute FBG values of overall female and their counterpart male mice during the 18 weeks are shown in (Table 2). Although each mouse line represents a distinct genotype, averaging the FBG values of 137 male mice and their line counterpart 143 female mice reveals a remarkable difference between the two sexes and the diets (Figure 1) The FBG values of both the 63 female and 76 male mice on CHD dropped significantly, with significant levels of *p* < 0.01 and *p* < 0.05, respectively, at 12 weeks compared with week 0 of the experiment, and continued to decrease significantly after 6 weeks of NDM treatment, with a significance level of *p* < 0.05, compared to w12 values. In contrast, the FBG values of the 61 male mice on HFD showed a non‐significant increase after 12 weeks, but a significant (*p* < 0.01) drop after NDM treatment. The FBG values of the 80 female mice on HFD show a non‐significant increase for 12 weeks as well as after NDM treatment.

**TABLE 2:** Total fasting blood glucose (FBG) (mg/dl) levels at the initial time point (week 0), week 12 and week 18 in females and males in both diet groups

week	FBG (mg/dl)			
	Females		Males	
	CHD	HFD	CHD	HFD
0	131.00 ± 3.90	125.88 ± 2.44	145.43 ± 3.91	151.16 ± 4.11
12	118.13 ± 2.93	130.29 ± 2.78	134.31 ± 4.64	156.25 ± 4.75
18	110.65 ± 3.66	133.58 ± 2.96	120.25 ± 3.64	139.11 ± 4.77

### 3.2 NDM affects male and female mice differently

**FIGURE 2:**
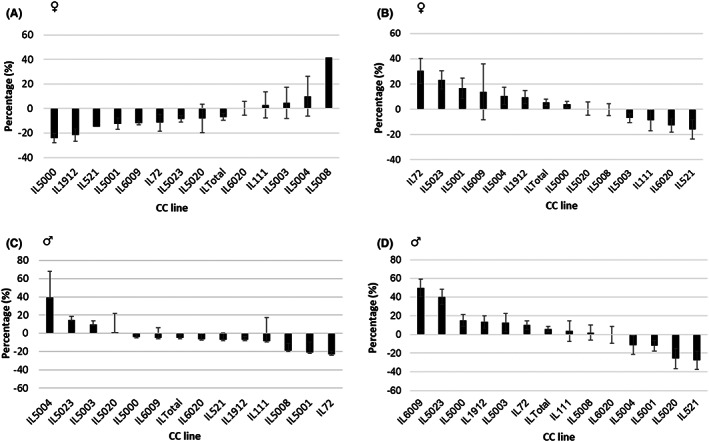
Percentage changes (Delta) in fasting blood glucose (FBG) between weeks 12 and 0 of the female and male mice of 13 different collaborative cross (CC) lines maintained on either a standard chow diet (CHD) or a high‐fat diet (HFD) for 12 weeks. Panels A and B show the profiles of the female mice of 13 different CC lines maintained on CHD and HFD, respectively, for 12 weeks. Panels C and D show the profiles of the male mice of 13 CC lines maintained on CHD and HFD, respectively, for 12 weeks. In all the panels A–D, the x‐axis shows the thirteen CC lines, while the y‐axis represents the percentage (%) FBG differences between weeks 12 and 0 during the experiment.

The data were further scrutinized by individual lines of mice on HFD for both female and male mice (Figure 2B,D, respectively). It is clear that the pattern of change in FBG values of the male lines is markedly different from the counterpart female lines, consistent with the differences in the average FBG values between the two sexes that were maintained on this diet.

### 3.3 NDM effects vary on CC lines with different diets

**FIGURE 3:**
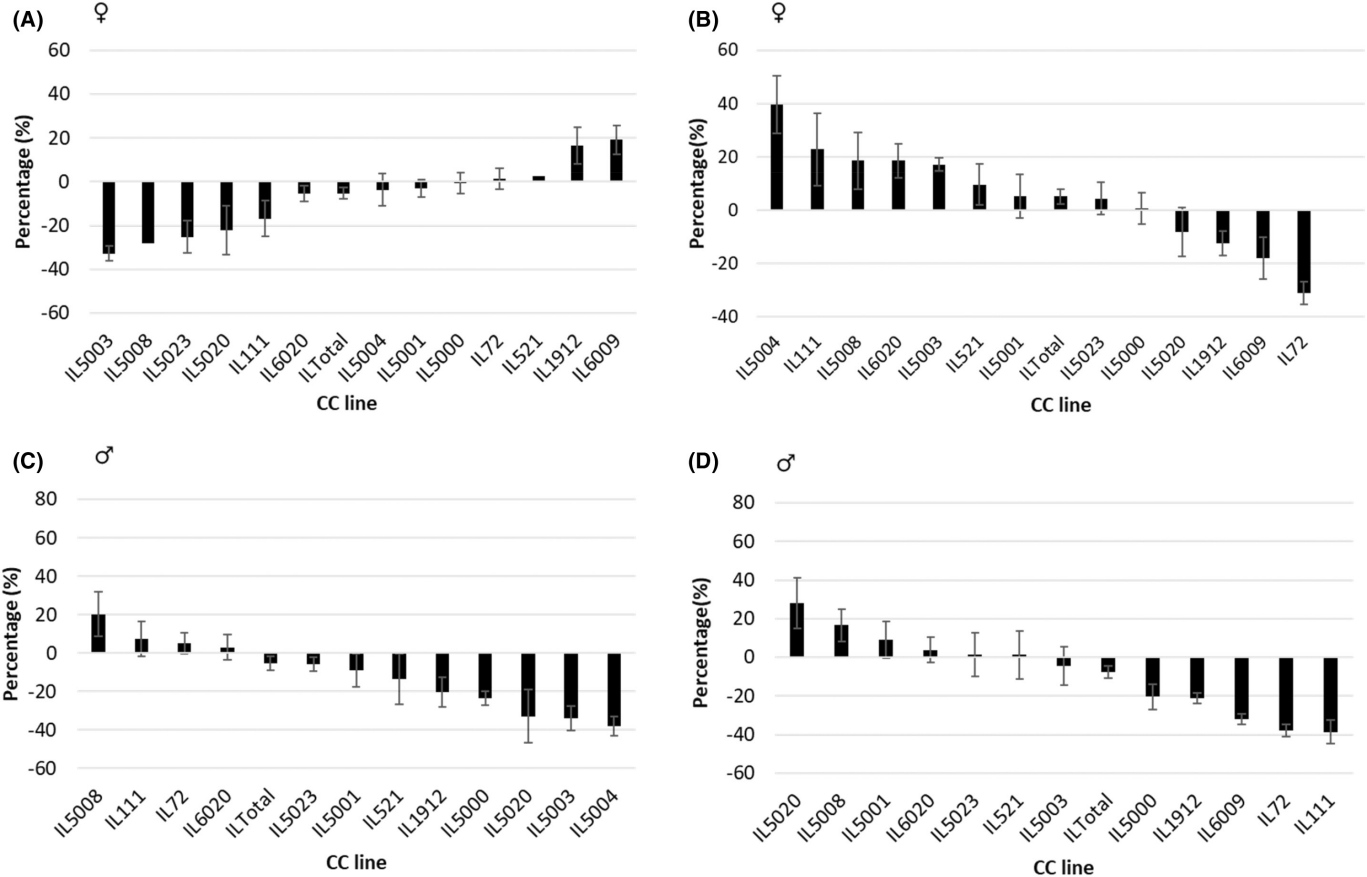
Percentage changes (Delta) in fasting blood glucose (FBG) between weeks 12 and 18 of the female and male mice of 13 different collaborative cross (CC) mice maintained on a standard chow diet (CHD) or a high‐fat diet (HFD) for weeks 12 and subsequently treated with NDM for six weeks. Panels A and B show the profiles of the female mice of 13 different CC lines maintained a CHD and HFD, respectively, during the 6 weeks of NDM treatment. Panels C and D show the profiles of the male mice of 13 CC lines maintained on a CHD and HFD, respectively, during the 6 weeks of NDM treatment. In all the panels A–D, the x‐axis shows the thirteen CC lines, while the y‐axis represents the percentage (%) FBG differences between weeks 12 and 18 during the experiment.

In contrast to the CC male lines, their female counterparts responded differently to the two diets and to the NDM treatment, as shown in Tables 3 and 4 for CHD and HFD, respectively. The mean FBG levels of the HFD fed and NDM treated females showed a non‐significant increase from a mean value of 130.29 ± 2.78 mg/dl at w12 to 133.58 mg/dl at w18, whereas during the same period of time in the CHD fed and NDM treated mice the mean value of the male FBG dropped significantly (Table 3). These remarkable sex‐associated differences between males and females of the 13 lines can be appreciated when the pattern of the NDM effects is presented in histograms, as shown in Figures 2 and 3.

**TABLE 3:** Effect of NDM on fasting blood glucose (FBG) levels in CC female mouse lines maintained on standard chow diet (CHD) for 12 weeks, and subsequently treated with NDM for six more weeks while maintained on the same diet. Summary of FBG (mg/dL) of female mice of 13 different of CC lines at age 8 weeks (w0 of the beginning of the experiment), at 12 weeks after maintaining on CHD (w12 + SE), changes in FBG (ΔFBG) between week 12 and week zero ((ΔFBG) (w0–12 + SE)) and %FBG changes between this period including standard error (% change ± SE) and indicating the significance status (^S^), FBG after 6 weeks treatment with NDM FBG (w18 ± SE), differences in %FBG changes (ΔFBG(w12–18 + SE)) between weeks 18 and 12, percentage of FBG changes between this period including standard error (% change ± SE) and indicating significant (S) or non‐significant (NS) status

Females	CHD group	FBG ± SE		Week 0 to 12			Week 12 to 18	
Line	No. of mice	Wk0	Wk12	ΔFBG	(% change ± SE)^s^	CRE FBG wk18 ± SE	ΔCRE FBG ± SE	(% change ± SE)^s^
IL72	5	146.00 ± 5.91	128.40 ± 8.12	−17.60 ± 10.35	(−11.45 ± 6.90)^ns^	128.80 ± 4.47	0.40 ± 5.77	(1.27 ± 4.76)^ns^
IL111	6	86.67 ± 5.84	89.17 ± 10.56	2.50 ± 9.33	(2.87 ± 10.58)^ns^	70.33 ± 4.45	−18.83 ± 7.87	(−16.83 ± 8.22)^ns^
IL521	1	137.00 ± 0.00	117.00 ± 0.00	−20.00 ± 0.00	14.6 ± 0.00	120.00 ± 0.00	3.00 ± 0.00	2.56 ± 0.00
IL1912	7	183.29 ± 9.78	141.86 ± 6.40	−41.43 ± 10.09	(−21.58 ± 4.97)^s^	163.14 ± 8.24	21.29 ± 10.57	(16.41 ± 8.38)^ns^
IL5000	5	145.40 ± 8.00	109.80 ± 6.76	−35.60 ± 6.47	(−24.13 ± 3.90)^s^	109.00 ± 8.19	−0.80 ± 5.11	(−0.66 ± 4.82)^ns^
IL5001	8	140.50 ± 4.79	121.88 ± 4.23	−18.63 ± 7.11	(−12.36 ± 4.76)^s^	117.50 ± 3.28	−4.38 ± 4.87	(−2.90 ± 3.97)^ns^
IL5003	4	132.00 ± 2.86	137.00 ± 13.87	5.00 ± 16.46	(4.54 ± 12.66)^ns^	90.75 ± 5.07	−46.25 ± 9.04	(−32.78 ± 3.46)^s^
IL5004	7	110.71 ± 12.67	110.71 ± 3.23	0.00 ± 10.84	(10.03 ± 16.29)^ns^	105.43 ± 5.85	−5.29 ± 8.46	(−3.66 ± 7.54)^ns^
IL5008	1	106.00 ± 0.00	150.00 ± 0.00	44.00 ± 0.00	41.51 ± 0.00	108.00 ± 0.00	−42.00 ± 0.00	(−28.00 ± 0.00)^ns^
IL5020	4	143.25 ± 6.52	134.00 ± 22.00	−9.25 ± 15.68	(−8.01 ± 11.60)^ns^	97.50 ± 8.74	−36.50 ± 19.48	(−22.29 ± 11.14)^ns^
IL5023	5	115.00 ± 4.91	105.00 ± 2.63	−10.00 ± 3.39	(−8.34 ± 2.63)^s^	78.20 ± 6.83	−26.80 ± 7.81	(−25.18 ± 7.52)^s^
IL6009	5	126.40 ± 6.04	111.80 ± 6.69	−14.60 ± 1.57	(−11.73 ± 1.48)^s^	132.20 ± 7.30	20.40 ± 7.04	(19.14 ± 6.67)^s^
IL6020	5	108.60 ± 5.94	107.60 ± 3.20	−1.00 ± 6.75	(0.16 ± 5.72)^ns^	101.40 ± 2.32	−6.20 ± 4.02	(−5.44 ± 3.43)^ns^
ILTotal	63	131.00 ± 3.90	118.13 ± 2.93	−12.87 ± 3.26	(−6.78 ± 2.85)^s^	110.65 ± 3.66	−7.48 ± 3.35	(−5.32 ± 2.64)^s^

**TABLE 4:** Effect of NDM on fasting blood glucose (FBG) levels in CC female mouse lines maintained on a high‐fat diet (HFD) for 12 weeks, and subsequently treated with NDM for six more weeks while maintained on the same diet. Summary of FBG (mg/dl) of female mice of 13 different of CC lines at age 8 weeks (w0 of the beginning of the experiment), at 12 weeks after maintaining on HFD (w12 + SE), changes in FBG (ΔFBG) between weeks 12 and week zero (ΔFBG) of (w0–12 + SE) and %FBG changes between this period including standard error (% change ± SE) and indicating the significant status (^S^), FBG after 6 weeks treatment with NDM FBG (w18 ± SE), differences in %FBG changes (ΔFBG (w12–18 + SE)) between weeks 18 and 12, percentage of FBG changes between this period including standard error (% change ± SE) and indicating the significant (S) or non‐significant (NS) status

Females	HFD group	FBG ± SE		Week 0–12		CRE FBG wk18 ± SE	Week 12–18	
Line	*n* = no of mice	Wk0	Wk12	ΔFBG	(% change ± SE)^s^		ΔCRE FBG ± SE	(% change ± SE)^s^
IL72	7	108.86 ± 5.34	138.86 ± 5.56	30.00 ± 9.57	(30.27 ± 9.96)^s^	94.57 ± 3.58	−44.29 ± 7.23	(−31.11 ± 4.29)^s^
IL111	5	93.60 ± 4.53	84.00 ± 3.54	−9.60 ± 7.67	(−8.71 ± 8.30)^ns^	102.00 ± 9.07	18.00 ± 10.49	(22.80 ± 13.64)^ns^
IL521	4	147.50 ± 8.51	122.00 ± 5.05	−25.50 ± 11.82	(−16.12 ± 7.65)^ns^	134.25 ± 13.33	12.25 ± 9.84	(9.58 ± 7.64)^ns^
IL1912	7	154.14 ± 6.30	167.14 ± 4.37	13.00 ± 7.96	(9.50 ± 5.29)^ns^	145.71 ± 6.29	−21.43 ± 7.86	(−12.43 ± 4.57)^s^
IL5000	4	140.50 ± 7.42	145.25 ± 4.33	4.75 ± 3.20	(3.75 ± 2.36)^ns^	145.50 ± 5.32	0.25 ± 8.58	(0.62 ± 5.90)^ns^
IL5001	11	124.09 ± 3.44	142.91 ± 8.65	18.82 ± 10.25	(16.57 ± 8.11)^ns^	145.27 ± 7.25	2.36 ± 9.75	(5.23 ± 8.09)^ns^
IL5003	9	144.11 ± 6.55	132.44 ± 4.03	−11.67 ± 4.86	(−7.08 ± 3.46)^ns^	154.56 ± 3.95	22.11 ± 2.83	(17.02 ± 2.47)^s^
IL5004	4	106.00 ± 5.40	116.00 ± 2.04	10.00 ± 7.07	(10.47 ± 7.00)^ns^	161.50 ± 10.99	45.50 ± 12.21	(39.61 ± 10.80)^s^
IL5008	5	112.80 ± 3.46	112.20 ± 5.45	−0.60 ± 5.24	(−0.39 ± 4.68)^ns^	131.40 ± 8.94	19.20 ± 11.51	(18.52 ± 10.65)^ns^
IL5020	5	133.80 ± 0.92	134.40 ± 7.11	0.60 ± 6.99	(0.44 ± 5.24)^ns^	121.60 ± 8.89	−12.80 ± 12.08	(−8.23 ± 9.20)^ns^
IL5023	7	110.57 ± 2.59	136.29 ± 8.63	25.71 ± 8.12	(23.27 ± 7.25)^s^	139.71 ± 6.19	3.43 ± 9.54	(4.33 ± 6.09)^ns^
IL6009	3	114.33 ± 9.24	126.33 ± 14.52	12.00 ± 22.59	(13.77 ± 22.24)^ns^	105.67 ± 22.02	−20.67 ± 7.80	(−18.03 ± 7.78)^ns^
IL6020	9	130.33 ± 6.99	110.89 ± 2.78	−19.44 ± 6.82	(−12.95 ± 5.19)^s^	131.11 ± 7.18	20.22 ± 6.91	(18.47 ± 6.54)^s^
ILTotal	80	125.88 ± 2.44	130.29 ± 2.78	4.41 ± 3.01	(5.42 ± 2.52)^s^	133.58 ± 2.96	3.29 ± 3.42	(5.16 ± 2.77)^ns^

Statistical analysis of the calculated %FBG data showed that overall significant changes in %FBG between w0 and w12 in the whole female mouse populations (Tables 3 and 4), but not in male mouse populations (Tables 5 and 6) maintained on HFD ranged between −16.12 to 30.27% and −27.81% to 50.00%, respectively. However, each individual line of the 13 mouse lines assessed performed differently. For the female mice on HFD, three out of the 13 lines (IL72, IL5023, and IL6020), and three male mouse lines (IL5000, IL5023, and IL6009) out of the assessed lines showed significant changes in the %FBG. After NDM treatment, overall male mouse populations showed significant changes in the %FBG, while no significant changes were noticed in females. Interestingly, in the CHD diet group, it was the opposite, i.e. after NDM treatment, the overall female mouse population showed significant changes in %FBG while no significant changes were noticed in males. Analysis of each CC mouse line, separately revealed that five female mouse lines (IL72, IL1912, IL5003, IL5004, and IL6020), as well as six male mouse lines (IL72, IL111, IL1912, IL5000, IL5004, and IL6009) showed significant changes in %FBG out of the 13 studied CC cohorts.

**TABLE 5:** Effect of NDM on fasting blood glucose (FBG) levels in CC male mouse lines maintained on standard chow diet (CHD) for 12 weeks, and subsequently treated with NDM for six more weeks while maintained on the same diet. Summary of FBG (mg/dl) of male mice of 13 different of CC lines at age 8 weeks (w0 of the beginning of the experiment), at 12 weeks after maintaining on CHD (w12 + SE), changes in FBG (ΔFBG) between weeks 12 and week zero ((ΔFBG) (w0–12 + SE)) and %FBG changes between this period including standard error (% change ± SE) and indicating the significant status ^(S)^, FBG after 6 weeks treatment with NDM FBG (w18 ± SE), differences in %FBG changes (ΔFBG (w12–18 + SE)) between weeks 18 and 12, percentage of FBG changes between this period including standard error (% change ± SE) and indicating the significant (S) or non‐significant (NS) status

Males	CHD group	FBG ± SE		Week 0 to 12		CRE FBG wk18 ± SE	Week 12 to 18	
Line	*n* = no of mice	Wk0	Wk12	ΔFBG	(% change ± SE)^s^		ΔCRE FBG ± SE	(% change ± SE)^s^
IL72	8	167.75 ± 3.00	128.38 ± 3.94	−39.38 ± 6.54	(−23.06 ± 3.65)^s^	134.13 ± 6.06	5.75 ± 7.07	(5.06 ± 5.59)^ns^
IL111	5	107.80 ± 14.68	84.20 ± 10.23	−23.60 ± 23.53	(−8.70 ± 25.64)^ns^	87.00 ± 5.01	2.80 ± 6.16	(7.30 ± 9.09)^ns^
IL521	3	120.67 ± 8.09	111.00 ± 4.58	−9.67 ± 10.17	(−7.10 ± 7.57)^ns^	97.00 ± 18.50	−14.00 ± 14.15	(−13.62 ± 13.15)^ns^
IL1912	4	197.75 ± 7.71	183.75 ± 11.84	−14.00 ± 7.82	(−7.17 ± 3.81)^ns^	145.00 ± 10.41	−38.75 ± 15.42	(−20.22 ± 7.63)^ns^
IL5000	3	146.67 ± 5.36	140.33 ± 2.85	−6.33 ± 4.06	(−4.15 ± 2.71)^ns^	107.33 ± 4.48	−33.00 ± 5.69	(−23.43 ± 3.78)^s^
IL5001	4	173.00 ± 7.60	134.25 ± 15.84	−38.75 ± 21.93	(−21.13 ± 11.44)^ns^	118.25 ± 5.17	−16.00 ± 12.81	(−8.98 ± 8.79)^ns^
IL5003	4	144.75 ± 4.13	158.25 ± 6.57	13.50 ± 6.01	(9.42 ± 4.30)^ns^	104.25 ± 9.89	−54.00 ± 11.16	(−33.86 ± 6.30)^s^
IL5004	3	129.00 ± 13.08	173.00 ± 19.97	44.00 ± 30.20	(39.13 ± 28.75)^ns^	107.00 ± 13.87	−66.00 ± 11.55	(−38.11 ± 4.96)^s^
IL5008	4	108.25 ± 5.91	86.75 ± 6.12	−21.50 ± 9.68	(−18.92 ± 7.90)^ns^	103.25 ± 8.40	16.50 ± 9.35	(20.25 ± 11.70)^ns^
IL5020	5	176.20 ± 12.78	168.60 ± 19.26	−7.60 ± 29.45	(1.09 ± 20.57)^ns^	103.20 ± 16.67	−65.40 ± 33.03	(−32.84 ± 13.72)^ns^
IL5023	4	141.75 ± 2.75	162.25 ± 4.50	20.50 ± 5.32	(14.59 ± 3.91)^s^	153.25 ± 8.56	−9.00 ± 5.61	(−5.69 ± 3.64)^ns^
IL6009	6	129.83 ± 9.36	121.83 ± 15.90	−8.00 ± 15.03	(−4.97 ± 10.90)^ns^	142.83 ± 12.21	21.00 ± 25.19	(27.59 ± 17.89)^ns^
IL6020	8	134.88 ± 4.34	124.63 ± 5.02	−10.25 ± 7.72	(−6.52 ± 6.22)^ns^	128.00 ± 8.93	3.38 ± 7.50	(2.92 ± 6.55)^ns^
ILTotal	61	145.43 ± 3.91	134.31 ± 4.64	−11.11 ± 4.73	(−5.14 ± 3.67)^ns^	120.25 ± 3.64	−14.07 ± 5.40	(−5.21 ± 3.62)^ns^

**TABLE 6:** Effect of NDM on fasting blood glucose (FBG) levels in CC male mouse lines maintained on a high‐fat diet (HFD) for 12 weeks, and subsequently treated with NDM for six more weeks while maintained on the same diet

Males	HFD group	FBG ± SE		Week 0–12		CRE FBG wk18 ± SE	Week 12–18	
Line	*n* = no of mice	Wk0	Wk12	ΔFBG	(% change ± SE)^s^		ΔCRE FBG ± SE	(% change ± SE)^s^
IL72	10	145.70 ± 2.52	159.90 ± 6.91	14.20 ± 6.69	(9.86 ± 4.62)^ns^	98.40 ± 4.84	−61.50 ± 6.75	(−37.90 ± 3.20)^s^
IL111	4	107.50 ± 6.61	109.50 ± 5.55	2.00 ± 10.46	(3.65 ± 11.09)^ns^	66.25 ± 3.84	−43.25 ± 8.05	(−38.74 ± 6.10)^s^
IL521	4	156.75 ± 9.25	110.50 ± 8.72	−46.25 ± 17.78	(−27.81 ± 9.81)^ns^	110.25 ± 12.15	−0.25 ± 14.51	(1.33 ± 12.38)^ns^
IL1912	9	182.33 ± 7.08	203.67 ± 6.78	21.33 ± 11.56	(13.33 ± 6.45)^ns^	159.78 ± 5.64	−43.89 ± 6.09	(−21.29 ± 2.58)^s^
IL5000	8	146.25 ± 4.64	166.75 ± 6.51	20.50 ± 8.44	(14.97 ± 6.45)^s^	131.00 ± 8.77	−35.75 ± 11.18	(−20.49 ± 6.44)^s^
IL5001	7	144.14 ± 2.40	127.14 ± 9.70	−17.00 ± 8.20	(−12.10 ± 5.63)^ns^	136.29 ± 11.08	9.14 ± 10.82	(9.12 ± 9.45)^ns^
IL5003	7	149.86 ± 8.68	167.71 ± 14.60	17.86 ± 13.42	(12.48 ± 9.75)^ns^	152.29 ± 4.94	−15.43 ± 14.19	(−4.43 ± 9.94)^ns^
IL5004	5	132.20 ± 13.75	112.20 ± 4.66	−20.00 ± 15.07	(−11.28 ± 9.86)^ns^	146.80 ± 5.55	34.60 ± 5.02	(31.31 ± 5.15)^s^
IL5008	4	122.75 ± 3.12	125.00 ± 10.59	2.25 ± 9.93	(1.78 ± 8.10)^ns^	143.25 ± 3.07	18.25 ± 10.18	(16.67 ± 8.34)^ns^
IL5020	5	241.80 ± 20.61	178.40 ± 27.09	−63.40 ± 26.18	(−25.64 ± 10.74)^ns^	218.60 ± 24.04	40.20 ± 19.68	(28.04 ± 13.09)^ns^
IL5023	4	143.00 ± 5.67	200.50 ± 15.11	57.50 ± 12.28	(40.10 ± 8.12)^s^	200.50 ± 17.73	0.00 ± 20.55	(1.46 ± 11.42)^ns^
IL6009	4	135.75 ± 10.62	201.25 ± 10.14	65.50 ± 9.31	(50.00 ± 9.40)^s^	136.25 ± 3.75	−65.00 ± 8.20	(−31.98 ± 2.60)^s^
IL6020	5	125.80 ± 13.60	121.00 ± 4.51	−4.80 ± 13.54	(−0.38 ± 9.00)^ns^	125.80 ± 9.28	4.80 ± 7.64	(3.87 ± 6.40)^ns^
ILTotal	76	151.16 ± 4.11	156.25 ± 4.75	5.09 ± 4.81	(5.60 ± 3.00)^ns^	139.11 ± 4.77	−17.14 ± 4.82	(−7.77 ± 3.22)^s^

*Note*: Summary of FBG (mg/dl) of male mice of 13 different of CC lines at age 8 weeks (w0 of the beginning of the experiment), at 12 weeks after maintaining on HFD (w12 + SE), changes in FBG (ΔFBG) between weeks 12 and week zero ((ΔFBG) (w0–12 + SE)) and %FBG changes between this period including standard error (% change ± SE) and indicating the significant status (^S^), FBG after 6 weeks treatment with NDM FBG (wk18 ± SE), differences in %FBG changes (ΔFBG (Wk12–18 + SE)) between weeks 18 and 12, percentage of FBG changes between this period including standard error (% change ± SE) and indicating the significant (S) or non‐significant (NS) status.

Statistical analysis of the overall %FBG – glycemic – levels of CHD‐fed male mice at w12 showed a non‐significant decrease compared to FBG levels at w0, and none of the mice exceeded 200 mg/dl of blood glucose (Table 5). During the following 6 weeks on the same diet and NDM treatment, the overall male population showed a non‐significant glycemic drop, while only three CC lines (IL5000, IL5003, IL5004) showed a significant drop in mean FBG levels from 134.31 ± 4.6 mg/dl at w12 to a mean FBG of 120.25 ± 3.64 mg/dl at w18 (Table 5) .

The HFD in male mice caused a significant increase in FBG at w12 in three lines (IL5000 IL5023, and IL6009) (Table 6). In mice kept on this diet and injected with NDM, a significant drop was seen in the FBG levels in six lines (IL72, IL111, IL1912, IL5000, IL5004 and IL6009), as well as in the mean FBG values of all lines from 156.25 ± 4.75 to 139.11 ± 4.77. Altogether, the data suggest that NDM treatment reduces FBG in some male CC lines irrespective of the diet.

### 3.4 Broad sense heritability and genetic coefficient of variation

The H2 and CV_g_ of the NDM effects on absolute and %FBG changes for females and males of the 13 mice lines maintained on CHD (control group) and HFD are shown in Table 7. H2 values were estimated for the FBG changes in experimental mice maintained on HFD before and after NDM treatment, and were found to be 0.60 and 0.50, respectively, for female mice and 0.64 and 0.73, respectively, for male mice. The estimated H2 for the FBG changes in control mice maintained on CHD before and after NDM treatment were found to be 0.39 and 0.80, respectively, for female mice and 0.63 and 0.43, respectively, for male mice (Table 7A). The CV_g_ values in mice maintained on HFD were 0.13 and 0.26 before and after NDM treatments, respectively, in female mice and 0.23 and 0.16, respectively, in male mice. Finally, the estimated H2 values for %FBG in control female and male mice maintained on CHD were 0.56 and 0.59, respectively, at w0, and the CV_g_ values were 0.16 and 0.18 of female and male mice, respectively (Table 7A).

**TABLE 7:** Broad sense heritability (H2) and genetic coefficient of variance (CVg) at the initial and pretreatment as well post‐treatment of NDM of the absolute (A) and percentage (B) FBG in female and male mice of 13 different CC lines at the initial time point of the experiment (FBG0), week 12 (pretreatment (FBG12)) and at week 18 weeks (post‐treatment (FBG18)) maintained on CHD or HFD and after treatment with NDM between weeks 12 and 18

A. Absolute FBG								
Time point	CHD (5% fat)				HFD (42% fat)			
	H2		CVg		H2		CVg	
	♀	♂	♀	♂	♀	♂	♀	♂
Initial	0.56	0.59	0.16	0.18	0.56	0.59	0.16	0.18
Pre‐treatment	0.39	0.63	0.13	0.23	0.60	0.64	0.16	0.23
Post‐treatment	0.80	0.43	0.26	0.16	0.50	0.73	0.15	0.28

B. % FBG								
Phenotype	CHD (5% fat)				HFD (42% fat)			
	H2		CVg		H2		CVg	
	♀	♂	♀	♂	♀	♂	♀	♂
% FBG 0–12	0.15	0.10	−1.3	−1.8	0.34	0.51	2.51	3.53
% FBG 12–18	0.47	0.43	−2.9	−3.8	0.46	0.63	3.43	−3.09

The estimated heritability and the CVg values were calculated for the %FBG changes for mice maintained on CHD or HFD during the first 12 weeks, as well as for the NDM treatment period (Table 7B). The calculated estimated heritability of %FBG over 0–12 weeks (%FBG 0–12) for female mice was 0.15 for mice maintained on CHD and 0.34 for mice maintained on HFD. The %FBG 12–18 was 0.47 of females on CHD and 0.46 for mice maintained on HFD. H^2^ values of %FBG 0–12 were 0.1 and 0.51 for male mice maintained on CHGD and HFD, respectively (Table 7B). H2 values of %FBG 12–18 for male mice were 0.43 for mice maintained on CHD and 0.63 for mice on HFD.

In female mice, the CV_g_ values of the %FBG 0–12 were −1.3 and 2.5 in mice maintained on CHD and HFD, respectively. CVg values of %FBG 12–18 were −2.9 and 3.34 in CHD and HFD, respectively.

The CVg values of %FBG 0–12 for male mice were −1.8 in CHD and 3.53 in HFD. For male mice, the CVg values of %FBG 12–18 were −3.8 for mice maintained on CHD and −3.09 for mice maintained on HFD.

The calculated heritability for absolute FBG values was found to be high, which indicates and confirms that these assessed traits are strongly influenced by host genetic background. Furthermore, the calculated CV_g_ shows that the genetic diversity is high in these tested 13 CC lines and is a promising resource for identifying genetic factors underlying these tested traits.

## 4 DISCUSSION

Previous studies employing HFD‐fed C57BI/6J male mice showed that cranberry extract treatments caused a beneficial effect on several metabolic health markers such as reversal of insulin resistance and glucose tolerance.^7^, ^35^, ^36^ Nevertheless, cranberry treatment of the C57BI/6J male mice did not lower fasting glycemia.^36^ In contrast, in the present study cranberry significantly lowered fasting glycemia in four (IL72, IL111, IL1912, and IL6009) out of the 13 male CC lines, suggesting that genetic background may be a crucial factor in this effect. These differences are demonstrable due to the unique CC lines employed in the present study as opposed to, for example, the single inbred mouse employed in the study of Anhe et al.^37^ Similar results were also observed in female mice of the CC lines, with two lines (IL72 and IL1912) showing a significant reduction in FBG after NDM treatment, while three different lines (IL5003, IL5004, and IL6020) showed a significant increase in FBG after NDM treatment.

Overall, regadrless of CC line and sex, the NDM treatment also produced a significant effect on both sexes of the assessed CC lines fed on CHD, however only three male mouse CC lines (IL5000, IL5003, and IL5004) showed a significant reduction of FBG after the treatment, while two female CC mouse lines (IL5003 and IL5023) showed a significant reduction in FBG and one line (IL6009) showed a significant increase in FBG. It is known that with the increase in genetic diversity and variations in mouse models, the possibility of capturing the diversity of phenotypes will increase. Interestingly, one of the studied CC lines (IL5004) showed a significant increase in FBG after NDM treatment.

The eight founders of the CC mouse population contain 42 million genetic variations (single nucleotide polymorphisms (SNPs)), which is higher than the human population (20 million SNPs).^16^, ^37^ Therefore, there is a strong case for the CC mouse population to be used for human studies to capture genes which may be translated to humans. The Collaborative Cross (CC) mouse model is a new and high genetically diverse reference population.^16^, ^38^ This unique model is a platform for research of complex traits that overcomes the limitation of existing animal models.^8^, ^9^, ^16^ The CC population is a powerful tool for genetic dissection of the different phenotypes. This large, multi‐parental, recombinant inbred strains panel exhibits recombinant chromosomal segments of approximately 2–5 Mb in size. The model was created by mating eight mouse strains, five classical inbred strains – A/J, 57BL/6J, 129S1/SvImJ, NOD/LtJ, NZO/H1LtJ, and three wild‐derived strains – CAST/EiJ, PWK/PhJ, and WSB/EiJ.^8^, ^9^, ^16^, ^39^ The eight founder strains capture a much greater level of genetic diversity than existing recombinant inbred lines (RILs) panels or other extant mouse genetic resource populations.^17^ Controlled randomization and minimization of selection during the breeding process recombined the natural genetic variation presented in these inbred strains. The result is a unique collection of RILs exhibiting a large phenotypic and genetic diversity that allows the tremendous genetic variation potential of these lines to be phenotypically expressed.^14^, ^16^ Full details of CC lines and their power of mapping quantitative trait loci (QTL) with host susceptibility to complex traits are presented in various publications.^8^, ^9^, ^10^, ^11^, ^12^, ^21^, ^30^, ^31^ The CC lines were generated by a breeding scheme in which the genomes of the eight founders were combined in three out‐breeding generations and then in inbreeding generations through sibling mating.^16^ Thus, each CC line generated from the original eight inbred mice represents a distinct population of mice with defined genetic backgrounds as opposed to a population of mice with diverse genetic backgrounds from one single inbred mouse strain. Calculation of the broad‐sense heritability and genetic coefficient of variation showed clearly that effects cranberry extract in lowering glucose are dependent on the host's genetic background. Furthermore, the genetic background required for the cranberry effect on a particular metabolic symptom (e.g. lowering glucose, as shown in the present study) is probably distinct from that required for a different symptom (e.g. reversing insulin resistance as shown by^7^, ^36^), emphasizing the power of employing the CC mice as opposed to inbred mice, which may or may not contain the genes required for the cranberry extract to exert its effect.

The results obtained in the current study have confirmed and agree with previously published results that have demonstrated the effect of high‐fat diet (HFD) on development of different severities of T2D and complications in CC mice, which are explained by host genetic diversity.^11^, ^20^, ^33^, ^36^ In the current study, we have demonstrated variations in the effect of NDM on reducing FBG, which is also explained by variations in host genetic background. These results also agree with our recent results showing the effect of NDM on reducing body weight, which is again explained by host genetic background.^29^

A drawback of the present study is the issue of dosage and mode of administration of the NDM. The dose of NDM (50 mg/kg mouse body weight) employed in the present study is ¼ of that employed in previous studies.^5^, ^34^ Little is known about the toxicity of the cranberry extracts but in none of the reports employing various extracts was a toxic effect noted. Moreover, if the effect on FBG in our experiments was due to toxicity, then we would expect it to occur in all mouse lines, which was not the case. Although the appropriate dose for use in humans is outside of the scope of the present study, a dose–response curve to determine the lowest dose of NDM will eventually be established for those lines bearing the genetic loci responsible for lowering blood glucose. Such a study should form the basis for determining doses in humans bearing the orthologous genetic loci.

In our study, we injected the NDM into the peritoneal cavity of the animals. This is a common technique in laboratory rodents but is rarely used in larger mammals and humans. Intraperitoneal (IP) injection is used for small species for which intravenous access is challenging and it can be used to administer large volumes of fluid safely or as a repository site for surgical implantation of a preloaded osmotic minipump. Although IP delivery is considered a parenteral route of administration, the pharmacokinetics of substances administered intraperitoneally are more similar to those seen after oral administration, because the primary route of absorption is into the mesenteric vessels, which drain into the portal vein and pass through the liver.^40^

Therefore, substances administered intraperitoneally may undergo hepatic metabolism before reaching the systemic circulation. In addition, a small amount of an intraperitoneally injected substance may pass directly across the diaphragm through small lacunae and into the thoracic lymph.^41^, ^42^

The route of administration is probably important, especially the oral route employed by Anhe et al.^7^, ^36^ Intraperitoneal delivery is different from the oral route, the former having the advantage of better controlling the cranberry dose administered. However, the route of administration is less important for defining whether the cranberry effect is or is not heritable, and once the genetic loci are defined, other routes of administration can be tested on more defined mouse lines and perhaps on humans carrying the orthologous loci. Furthermore, IP delivery is considered a parenteral route of administration and the pharmacokinetics of substances administered intraperitoneally are more similar to those seen after oral administration because the primary route of absorption is into the mesenteric vessels, which drain into the portal vein and pass through the host organs.

In this study, IP injections were used to administer the NDM to the assessed mice. However, in both routes of administration (IP injection and oral feeding), drugs enter the systemic circulation mostly through the hepatic portal system. The only difference is in the absorption phase; with oral administration, the substances are absorbed in the gastrointestinal tract, and with IP injection, they are diffused across the peritoneal membrane, which is a semipermeable membrane, lined with a capillary bed. The blood vessels supplying and draining the abdominal viscera, musculature, and mesentery constitute a blood‐filled compartment into which drugs can diffuse from the peritoneum. Absorption of intraperitoneally applied drugs occurs rather rapidly but compounds are partially subjected to hepatic first‐pass elimination. The effect of biopharmaceutical and biological factors on drug bioavailability is known only to a limited extent. Intraperitoneal administration will have more bioavailability as it is administered directly into the circulation. This leads to an increased absorption rate of the substance after IP administration.^43^ However, one limitation of this route is the first pass metabolism, as in the orally administered drugs, because substances absorbed from the peritoneal cavity end up in the portal vein and pass through the liver.^40^, ^43^, ^44^

The dose of NDM (50 mg/kg mouse body weight) used in our study was based on previous reports^7^, ^14^ showing that cranberry extract contains bioactive components and has a significant effect on oral inflammation. Therefore, in this study, we adopted this dose, which indeed showed an active effect on body weight loss, depending on the host genetic background. Furthermore, in previously published papers, the usual amount of whole cranberry extract administered was 200 mg/kg mouse body weight, which is fourfold what we used in our study.^13^, ^36^ Our NDM cranberry fraction (shown in previous studies to consist of components active against inflammation) is only part of the whole cranberry extract, which was used in previous studies.^13^, ^14^ Therefore, we used ¼ of the amount of cranberry extract normally used in previous studies, to avoid any overdose effects and observe any unexpected reactions.

Toxicity effects are a major concern when administering herbal and dietary supplements. Little is known about the toxicity of cranberry extracts but in none of the reports employing various extracts was a toxic effect noted, even in studies where fourfold (i.e. 200 mg/kg mouse body weight) the dosage we used was employed.^13^, ^14^ Although the appropriate dose to be used in humans is outside of the scope of the present study, a dose–response curve to determine the lowest dose of NDM will eventually be determined on those lines bearing the genetic loci responsible for lowering FBG. Such a study should form the basis for determining dosing in humans bearing the orthologous genetic loci. Interestingly, this effect can be vary with different host genetic backgrounds (inter‐individual toxicity responses), as previously reported.^45^ In that study, Church and colleagues showed that within the diversity outcross (DO) population, equal exposure to green tea extract containing the polyphenol epigallocatechin gallate (EGCG; 50 mg/kg daily for 3 days) was found to be tolerated without hepatotoxicity in the majority of mice; however, a small fraction of the animals (16%; 43/272) exhibited severe hepatotoxicity (10%–86.8% liver necrosis), which was assumed to be analogous to clinical cases. In our study, the toxicity effect of NDM was not assessed, because a previous study^46^ reported that at an amount of 4 mg per day (160 mg/kg mouse body weight for an average mouse weight of 25 g) toxicity was not observed. However, fourfold this amount i.e. 16 g of NDM (640 mg/kg mouse body weight for an average mouse weight of 25 g) was toxic. Although we employed body weight measurements only in the present study, we realize that metabolic syndrome includes many other metabolic parameters such as fat distribution, liver weight, determination of serum components such as adiponectin, leptin, and ghrelin, fasting blood glucose, and glucose tolerance (insulin resistance), as well as changes in the microbiome. Indeed, studies are being designed to include some of these parameters in the CC mice to better understand the effect of cranberry extracts on metabolic syndrome.

The average person can drink one or two glasses per day of cranberry juice cocktail (e.g. 400–600 ml, which nutritionally also treats urinary tract infection (UTI), for example). This amount contains 0.2 mg NDM/ml juice, meaning that 1.07–1.33 mg/kg NDM per day at the most is consumed by an adult man (weighing 75 kg on average). If so, the amount we injected is equivalent to the amount of NDM in 2 L juice consumed per day by the average man. Therefore, using NDM (4 × 1.07–1.33 = 4.28–5.32 mg NDM/kg per day for humans) could reduce the volume of whole cranberry juice required. This agrees with the United States FDA recommendations regarding the calculation for converting a daily dosage found to be effective in mouse models into a human equivalent dosage, which should be 0.08 of what is used in the mouse model. That is, 0.08 of 50 mg/kg dose in a mouse model, equal to 4 mg/kg in humans, may be used.

The results show the reduction in FBG of mice maintained on HFD in 13 CC mice lines following the IP administration of NDM‐CRE is a heritable trait. The high estimated broad‐sense heritability (H2) shows the importance of the host genetic background in the performance of these assessed traits, and genetic covariance (CVg) indicates that we have a strong genetic diversity in the assessed population that will lead to identifying a variety of factors underlying these traits. The 13 CC strains, which were randomly used in this study, are part of the currently available large CC population (75 lines), which constitutes a powerful mouse genetic reference population (GRP) for gene mapping and dissecting complex traits. At this stage, 13 CC lines were assessed for the effects of the NDM from cranberry extract, just to determine if the host genetic background defines the efficacy of the response to the treatment, which indeed was confirmed in our study. After obtaining these results, we plan to continue the breeding of CC lines for further assessment of NDM treatment, so as to provide enough power to perform gene mapping. The advantage of the CC lines compared with the eight parental founders is that the new genetic (gene–gene) interactions of the eight genomes together in the CC lines may generate new epistatic structures that should perform better than the parental lines alone.

The biologically active components of NDM are oligomers of cathechin and epicathechin composed of 3–6 degrees of polymerization corresponding to a range of 886–1728 MW.^14^ These polyphenolic polymers form stable microaggragates in aqueous or 50% ethanol solutions. Such microaggregates are retained in dialysis tubes with a 12 000–14 000 MW cutoff, and hence are referred to as ‘high MW’. Because it was found in a number of studies that following ingestion of cranberry juice or powder by rats^47^, ^48^ and humans^49^, ^50^ catechin subunits compounds are secreted in urine, it is likely that the oligomeric cathechin is absorbed by the epithelial barrier and degraded to biologically active phenolic compounds by internal organs such as the liver. In fact, it has been argued that although the metabolism of phenolic compounds in vivo is complex and poorly understood,^51^ they are degraded by the liver and other organs. We hypothesize that a similar fate of adsorption and degradation of phenolic oligomers in the intraperitoneal cavity may occur. Certainly these molecules can be adsorbed by the epithelium barrier to enter the gut and then reabsorbed to reach the internal organs, as occurs with the ingestion of such compounds. It follows that the extent of adoption and degradation of the polyphenolic molecules is dependent on a particular genetic background, which explains the variability of the effect of NDM in the CC lines.

In conclusion, our study shows an effect of cranberry extract in lowering fasting blood glucose levels and that this effect is dependent on the host's sex (pharmacosex), genetic background (pharmacogenetics), and diet type, i.e. CHD or HFD (pharmacodiet). Because the DNA sequence of both the mouse founders and the CC lines are known, there is no doubt that the genetic loci or genes involved in such an effect may be determined once more CC lines of mice are employed. This will allow identification and use of mouse lines showing the maximum response to NDM to eventually determining the appropriate dosage and route of administration of the extract in humans. All these results support the need for further research to better understand and implement a personalized medicine approach/utilization of NDM for reducing FBG levels.

Finally, the results of our study have shown that amount of NDM we used induces a significant effect, and this is believed to be due to the high purity and concentration of the active component in our NDM extract.

## AUTHOR CONTRIBUTIONS

Fatima Amer was involved in project design, execution, data analysis, and MS preparation, Rana Tarabeih was involved in project execution, and Itzhak Ofek was involved in project design, data analysis, and MS preparation. Fuad A. Iraqi was involved in the project design, data analysis, and MS preparation and approving its final version.

## CONFLICT OF INTEREST

None.

## ETHICS STATEMENT

All experimental protocols proposed to be used in this project approved by the Institutional Animal Care and Use Committee (IACUC) (Approval number‐01‐17‐056), which adherers to the Israeli guidelines which follows the NIH/USA animal care and use protocols. Proposed mice to be sued are monitored daily by the student for their overall health status. Mice, which were observed to suffer (less movement and activity) from the infection, and based on the consultation with the Veterinarian at the small animal unit, were terminated. Furthermore, based on the approved protocol, mice which lost 10% of their bodyweight between two measured points, or 20% overall, of their initial bodyweight, were also terminated. Termination procedure of mice during assessment based animal suffering or at endpoint of the experiment was based of injection of Ketamine and Xylazine with dose of 100 mg/kg (body weight) and 10 mg/kg (bodyweight), respectively for initial euthanasia, and subsequently by CO_2_ chamber.
